# A Prospective Cohort Study on Cardiotoxicity of Adjuvant Trastuzumab Therapy in Breast Cancer Patients

**DOI:** 10.5935/abc.20160084

**Published:** 2016-07

**Authors:** Erika Matos, Borut Jug, Rok Blagus, Branko Zakotnik

**Affiliations:** 1 Institute of Oncology Ljubljana - Division of Medical Oncology - Slovenia; 2 University Medical Centre Ljubljana - Department of Vascular Diseases - Slovenia; 3 Institute for Biostatistics and Medical Informatics - University of Ljubljana - Slovenia

**Keywords:** Trastuzumab / adverse effects, Trastuzumab / therapeutic use, Breast Neoplasms / therapy, Cardiotoxicity, Cohort Studies

## Abstract

**Background:**

Cardiotoxicity is an important side effect of trastuzumab therapy and cardiac surveillance is recommended.

**Objectives:**

The aim of our study was to prospectively assess baseline patients' characteristics, level of N-terminal pro-brain natriuretic peptide (NT-proBNP) and echocardiographic parameters as possible predictors of trastuzumab-related cardiac dysfunction.

**Methods:**

In a prospective cohort study, clinical, echocardiographic and neurohumoral assessment was performed at baseline, after 4, 8 and 12 months in breast cancer patients undergoing post-anthracycline (3-4 cycles) adjuvant therapy with trastuzumab. Trastuzumab-related cardiac dysfunction was defined as a decline of ≥ 10% in left ventricular ejection fraction (LVEF).

**Results:**

92 patients (mean age, 53.6 ± 9.0 years) were included. Patients who developed trastuzumab-related LVEF decline ≥ 10% (20.6%) during treatment had significantly higher baseline LVEF (70.7 ± 4.4%) than those without (64.8 ± 5.5%) (p = 0.0035). All other measured baseline parameters (age, body mass index, arterial hypertension, level of NT-proBNP and other echocardiographic parameters) were not identified as significant.

**Conclusions:**

Our findings suggest that baseline patient' characteristics, level of NT-proBNP and echocardiographic parameters, as long as they are within normal range, are not a reliable tool to predict early trastuzumab-related cardiac dysfunction in patients undergoing post-low dose anthracycline adjuvant trastuzumab therapy. A LVEF decline in patients with high-normal baseline level although statistically significant is not clinically relevant.

## Introduction

Adjuvant trastuzumab significantly reduces mortality and the risk of relapse in human epidermal growth factor receptor-2 (HER-2) positive breast cancer patients.^1-6^ However, trastuzumab therapy is associated with significant cardiotoxicity and trastuzumab-related cardiac dysfunction has been recognized as an important side effect and the main reason for premature therapy discontinuation.^1,7^

High blood pressure (HBP), age over 50 years, increased body mass index (BMI), exposure to anthracyclines and borderline baseline left ventricular ejection fraction (LVEF) are known risk factors for trastuzumab-related cardiotoxicity, derived from randomized adjuvant trials.^8^ Regarding the other possible predictive factors for trastuzumab-related cardiotoxicity, such as N-terminal pro-brain natriuretic peptide (NT-proBNP)^9-11^ and some echocardiographic measurements, like left ventricular end-systolic volume (LVESV), peak systolic wave velocity at septal mitral position in tissue Doppler imaging (Sm wave), early diastolic wave velocity at septal mitral position in tissue Doppler imaging (Em wave), left atrial area (LA) and E to A wave velocity ratio in mitral inflow pulse Doppler (E/A), either evidence is weak or have not been studied extensively.^12-15^ It is known that deterioration of LVEF is a relatively late stage of ventricular dysfunction, as the myocardium has exhausted its considerable functional reserve. However, diastolic impairment of left ventricle occurs and can be echocardiographically demonstrated before the fall in LVEF in different pathologies, like anthracycline-mediated cardiotoxicity, coronary artery disease, diabetes, or HBP. Therefore, to demonstrate the earliest and subtle cardiac changes during adjuvant trastuzumab treatment looking at diastolic function seems reasonable. The currently available data in this patient population are scarce and not uniform.^15-18^ Natriuretic peptides, released during hemodynamic stress, are widely used in the early detection of heart failure and have already been shown to be sensitive markers of left ventricular dysfunction and powerful markers of morbidity and mortality in heart failure setting. Increased levels of NT-proBNP have also been detected in some studies evaluating cardiotoxicity due to anthracycline treatment. However, the ability of NT-proBNP to predict early cardiac dysfunction in trastuzumab-treated patients remains unconfirmed with most studies yielding disappointing results.^11,13,19^

Previous studies reported that changes in serial echocardiographic measurements (especially in the first three months after therapy initiation) predict trastuzumab-related cardiac dysfunction. However, they did not focus on possible predictive value of baseline measurements derived by two-dimensional Doppler echocardiography and pulse wave tissue Doppler imaging.^13,20^ Studies have demonstrated that two-dimensional echocardiographic techniques are reliable in the detection of difference in LVEF close to 10% and therefore can be used as an indicator of cardiotoxicity in the absence of symptoms.^21^

In the present prospective study, we assessed age, HBP, BMI, LVEF, LVESV, Sm and Em wave, LA, E/A and NT-proBNP level as possible predictors of trastuzumab-related cardiac dysfunction in HER-2 positive breast cancer patients undergoing post-anthracycline adjuvant trastuzumab therapy.

## Methods

This was a prospective cohort study with serial clinical, echocardiographic and neurohumoral assessment in HER-2 positive breast cancer patients undergoing adjuvant therapy with trastuzumab at the Institute of Oncology in Ljubljana, Slovenia, from October 2011 to November 2013. The study was approved by the National Ethics Committee (No. 110/04/10) and all participants gave informed consent prior to study entry.

Patients were considered eligible if baseline systolic cardiac function was normal (LVEF > 50%). All patients were pretreated with anthracycline-based adjuvant chemotherapy (3-4 cycles). Besides anthracyclines, taxane-based adjuvant chemotherapy was given according to current international guidelines, concomitantly with trastuzumab.^22^ Patients were treated with trastuzumab for one year, altogether 18 infusions every three weeks. Radiotherapy to the breast or mammary region was delivered after adjuvant chemotherapy, when on treatment with adjuvant trastuzumab, if indicated according to current clinical guidelines.^22^

At baseline, and after 4, 8 and 12 months during trastuzumab treatment, clinical examination and comprehensive transthoracic echocardiography were performed, and plasma levels of NT-proBNP were determined.

Patients underwent thorough cardiovascular evaluation and were diagnosed with heart failure in the presence of signs and symptoms, and structural heart disease according to the European Society of Cardiology guidelines.^23^

Regarding co-morbidities, we were specially focused on HBP, dyslipidaemia and diabetes mellitus. High blood pressure was defined as history of arterial hypertension with appropriate antihypertensive medical management, or as a > 140 mmHg systolic and/or > 90 mmHg diastolic blood pressure on two separate measurements. Dyslipidaemia and diabetes mellitus were defined as history of either disease with appropriate medical management, or as laboratory findings (LDL-C > 3, TC > 5 mmol/L or fasting blood glucose level > 7 mmol/L, respectively). Body mass index was calculated based on the following formula: body weight in kilograms divided by height in meters squared. Coronary artery disease was defined as history of myocardial infarction or revascularization procedures, presence of coronary stenosis on invasive or computed tomographic angiography or presence of ischemia on myocardial perfusion imaging. Valvulopathy was defined as a history of valvular repair/replacement, or by significant (> mild) structural or functional valve impairment on cardiac imaging.

Transthoracic echocardiography was performed with a phased-array imaging system equipped with a transducer with second harmonics capability. Images were obtained in the parasternal long- and short-axis and apical views, with the subject lying in the left lateral position. Tissue Doppler imaging, standard M-mode, two-dimensional Doppler and pulse wave tissue Doppler recordings were acquired. All measurements were made according to the recommendations of the American Society of Echocardiography (ASE) and the European Association of Echocardiography (EAE).^18,24^ Each assessment was analysed for at least three consecutive cycles (or at least five in any rhythm other than regular sinus rhythm), avoiding post-ectopic beats. Left ventricular ejection fraction was assessed on two-dimensional apical four- and two-chamber views, using the biplane Simpson method. If biplane apical views were not available at baseline and/or follow-up, the analysis was conducted using the apical four-chamber view. No echocardiographic measurement was excluded from the study because of poor image quality. Transmitral pulsed Doppler was recorded in the apical four-chamber view in order to measure early (E) and atrial (A) peak velocities (m/s), peak velocity *E*/A ratio and E velocity deceleration time (ms). Pulsed tissue Doppler was performed at the level of the lateral mitral annulus and septal mitral annulus in order to measure peak systolic (Sm), and early (Em) and late (Am) diastolic velocities. All tissue Doppler parameters were obtained as the average of the values of the lateral and septal mitral annulus. The ratio of Doppler transmitral E peak velocity and average *Em* peak velocity (lateral Em + septal Em/2) was calculated as an index of left ventricular filling pressure.

All echocardiographic evaluations were performed by one experienced cardiologist in one institution. Method reproducibility was assessed at the echocardiography laboratory using 20 recordings analysed twice (intra-reader variability); intra-class correlation coefficient was good (for LVEF intra-reader, 0.97). Method reliability was assessed by comparing LVEF derived from echocardiography with multigated acquisition scan (MUGA) in 20 patients; inter-class correlation coefficient was good (for LVEF inter-method, 0.87).

Serum levels of NT-proBNP were determined by electrochemiluminescence immunoassay (ECLIA) on Cobas e411 analyser (Roche Diagnostics GmbH, Mannheim, Germany).

Trastuzumab-related cardiac dysfunction was defined as clinical signs and/or symptoms of heart failure or a decline in LVEF of at least 10% from baseline in asymptomatic patients throughout the adjuvant trastuzumab treatment.^3,16^

According to the literature, the expected incidence of LVEF decline of 10% or more in patients on trastuzumab adjuvant therapy was 15%.^16^ For a desired probability level of 0.05 and statistical power of 0.80 to detect a 10% decline in the range of normal LVEF values (70% to 60%), we needed 12 patients with this feature, thus we have calculated that we needed to include 90 patients.

Normally distributed continuous variables were described as mean ± standard deviation (SD), non-normally distributed continuous variables were described as median and interquartile range and categorical variables were described as numbers and/or percentages. Baseline characteristics and echocardiographic parameters of patients with and without trastuzumab-related cardiac dysfunction at follow-up were compared with logistic regression; odds ratios (OR) and their 95% confidence intervals (CI) are reported. Multiple logistic regression analysis was performed to determine the independent predictors for a decline in LVEF of at least 10% from baseline using the significant univariate predictors. A two-tailed p value < 0.05 was considered significant. Goodness-of-fit of the models was estimated with Akaike information criterion (AIC)^25^ and Hosmer-Lemeshow goodness-of-fit test;^26^ none of the models reported in this paper exhibited a statistically significant misfit. Analyses were performed using IBM SPSS v.22 software and R language for statistical computing (R v.3.0.3.).^27^

## Results

A total of 92 patients gave informed consent and were included. None of the patients who gave informed consent was lost to follow-up and none of the patients died. One patient was excluded from analysis because her baseline echocardiography (post-anthracycline) suggested right ventricular overload and suspected pulmonary embolism, which was confirmed by CT angiography. All the remaining patients received the whole one year of adjuvant trastuzumab by plan. There were no temporary discontinuations from the therapy. All patients were female with a mean age of 53.6 years (range 35.0-75.5). All were pretreated with anthracyclines (3 or 4 cycles of epidoxorubicin 90 to 100 mg/m^2^ or 4 cycles of doxorubicin 60 mg/m^2^) and 82 (89.1%) patients received also taxane-based chemotherapy. In total, there were 288 echocardiographic measurements and 297 NT-proBNP measurements. Left ventricular ejection fraction decline has been determined for 78 patients, who have had both, baseline and at least one additional measurement. Certain echocardiographic parameters could not be assessed at baseline for all patients.

**Figure 1:**
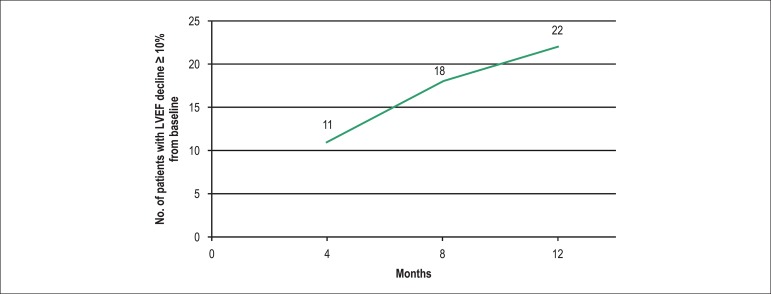
Incidence of cardiotoxicity during trastuzumab treatment.

Sixty-five (70.7%) patients had radiotherapy and 60 (65.2%) patients had adjuvant endocrine therapy concomitantly with trastuzumab. Co-morbidities were recorded from patients' charts: HBP in 27 (29.3%) patients, diabetes mellitus in 4 (4.3%) patients, and dyslipidaemia in 9 (9.8%) patients. There were 25 patients treated for HBP before study entry. Out of them, 12 patients were treated with monotherapy: ACE inhibitor (5 patients), angiotensin receptor blocker (2 patients), beta-blocker (4 patients), and calcium channel blocker (1 patient). In the remaining 13 patients, HBP was treated with the combination of two or three drugs, one being most commonly diuretic, and the other: ACE inhibitor (5 patients), angiotensin receptor blocker (3 patients), ACE inhibitor and beta-blocker (2 patients), ACE inhibitor and calcium channel blocker (2 patients). One patient was receiving inappropriate combination of ACE inhibitor and angiotensin receptor blocker and this has been modified during the treatment. In 2 patients with known history of HBP (both of them were refusing treatment before), therapy with ACE inhibitor was introduced. Detailed patients and treatment characteristics are summarized in Table 1. None of the patients developed symptomatic heart failure during follow-up; however, echocardiographic assessment revealed a decline of LVEF (≥ 10%) during treatment in 22 (23,9%) patients. Out of these, 11 (50%) experienced a decline during the first 4 months since baseline, 7 (31.8%) between 4 and 8 months since baseline, and 4 (18.2%) between 8 and 12 months since baseline (Figure 1). All these patients were additionally referred to a cardiologist for follow-up during treatment.

**Table 1:** Patients and treatment characteristics

**No.**	**92**	**100%**
**Age / years**		
Mean (SD)	53.6 (9.0)	
Range	35.0-75.5	
**Breast cancer side**		
Left	46	50%
Right	46	50%
**BMI / kg/m^2^**		
Mean (SD)	26.1 (4.9)	
Range	17.9-40.1	
No. of patients with BMI 25-29.9 kg/m^2^	30	32.6%
No. of patients with BMI ≥ 30 kg/m^2^	16	17.4%
**Co-morbidities**		
HBP	27	29.3%
DM	4	4.3%
Dyslipidaemia	9	9.8%
**Treatment**		
Cumulative dose of anthracycline		
Doxorubicin (mean) / mg/m^2^	240	10.9%
Epidoxorubicin (mean) / mg/m^2^	304	89.1%
RT	65	70.7%
ET	60	65.2%
Taxane-based chemotherapy	82	89.1%

BMI: body mass index; HBP: high blood pressure; DM: diabetes mellitus; RT: radiotherapy; ET: endocrine therapy; SD: standard deviation.

In the univariate model only baseline LVEF and LVESV were found to predict significant decline in LVEF of 10% or more during adjuvant trastuzumab treatment (Table 2). The mean baseline LVEF was 70.7% and 64.8% for those with and those without trastuzumab-related cardiac dysfunction. The difference for LVEF as well as for LVESV was statistically significant; in multivariate analysis, only LVEF retained statistical significance (Table 3). There was no significant impact of age, BMI, HBP, baseline NT-proBNP level between patients with and without significant decline in LVEF. None of the Doppler ultrasound and tissue Doppler derived parameters of systolic or diastolic function approached statistical significance (Table 2).

**Table 2:** Baseline characteristics and echocardiographic parameters of patients with and without trastuzumab-related cardiac dysfunction at follow-up – univariate model

**Parameter**	**All patients**	**LVEF decline ≥ 10% [No.]**	**LVEF decline < 10% [No.]**	**OR**	**95% CI**	**p**
Age (years)	53.6 ± 9.0	53.2 ± 9.4 [22]	52.6 ± 9.0 [56]	1.008	0.954 – 1.065	0.7753
BMI (kg/m^2^)	26.1 ± 4.9	25.2 ± 5.2 [22]	26.5 ± 5.4 [56]	1.001	0.902 – 1.112	0.9805
LVESV (ml)	21.8 ± 8.0	17.8 ± 5.0 [22]	22.6 ± 6.6 [56]	0.858	0.774 – 0.950	0.0033
LVEF (%)	66.3 ± 5.8	70.7 ± 4.4 [22]	64.8 ± 5.5 [56]	1.284	1.128 – 1.462	0.0002
Sm (cm/s)	8.5 ± 1.8	8.1 ± 1.9 [22]	8.6 ± 1.8 [54]	0.945	0.717 – 1.242	0.6847
NT-proBNP (pg/ml)	79 (45-133)	113 (61–165) [22]	83 (53–114) [54]	1.003	0.996 – 1.009	0.3920
Em (cm/s)	8.5 ± 2.7	8.4 ± 2.5 [22]	8.5 ± 2.8 [54]	1.000	0.834 – 1.200	0.9992
LA (cm^2^)	15.8 ± 3.4	15.6 ± 2.9 [22]	16.1 ± 3.1 [51]	0.945	0.800 – 1.116	0.5067
E/A	1.0 ± 0.3	1.0 ± 0.3 [22]	0.99 ± 0.2 [52]	1.194	0.226 – 6.299	0.8349
HBP (%)*	27/92 (29.3%)	7/17 (41.1%) (22]	20/75 (26.7%) [56]	1.892	0.672 – 5.329	0.2273

* Prevalence of hypertension is presented as number (proportion) of patients. NT-proBNP levels are presented as median and interquartile range. All other data are presented as mean ± SD.

LVEF: left ventricular ejection fraction(Simpson biplane method); BMI: body mass index; LVESV: left ventricular end systolic volume; Sm: peak systolic wave velocity at septal mitral position in tissue Doppler imaging; Em: early diastolic wave velocity at septal mitral position in tissue Doppler imaging; LA: left atrial area; E/A: E to A wave velocity ratio in mitral inflow pulse Doppler; HBP: high blood pressure; NT-proBNP: N-terminal pro-brain natriuretic peptide; OR: odds ratio; No.: number of patients.

**Table 3:** Multivariate predictors of trastuzumab-related cardiac dysfunction

**Parameter**	**Estimate**	**SE**	**OR**	**95% CI**	**p**
Intercept	-15.0019	5.8521	0.000	0.000 – 0.029	0.0104
LVEF (%l)	0.2207	0.0755	1.247	1.075 – 1.446	0.0035
LVESV (ml)	-0.0422	0.0589	0.959	0.854 – 1.076	0.4739

LVEF: left ventricular ejection fraction; LVESV: left ventricular end systolic volume; SE: Standard Error; OR: odds ratio.

## Discussion

In the present study, we found that classic risk factors (age, HBP, BMI), NT-proBNP, echocardiographic measurements (Sm and Em wave, LA, E/A) are not predictors of trastuzumab-related left ventricular dysfunction in HER-2 positive breast cancer patients undergoing post-low dose anthracycline adjuvant trastuzumab therapy. Patients with higher baseline LVEF values (70.7% vs 64.8%) were found to have a statistically significant but clinically irrelevant LVEF decline of 10% or more during treatment.

The age, incidence of HBP and BMI of our patients were comparable to reported adjuvant studies.^2,4,5^ In our study patients' age was not found as an important predictive factor, which is in contrary to the results of both North American prospective randomized clinical studies.^2,5^ On the other hand, there are studies that could not confirm its predictive value, among them the HERA study.^4^ In addition, some studies including patients from daily clinical practice could not demonstrate the impact of patients' age in predicting cardiac dysfunction when on trastuzumab adjuvant treatment.^14,28^ High blood pressure, the most extensively studied co-morbidity in this patient population, is a generally accepted risk factor and its importance was confirmed in several studies.^5,29^ But the results are not uniform and there are also studies that could, like ours, not demonstrate its predictive role.^2,30,31^ In the HERA study,^2^ BMI was found to be an important risk factor, however this was not demonstrated or not extensively studied in another two randomized studies.^5,29^ In our study, BMI did not emerge as important.

The majority of studies aimed to demonstrate the utility of NT-proBNP level in predicting trastuzumab cardiotoxicity were disappointing.^10,19,32^ However, due to its wide availability and low cost, NT-proBNP is still very attractive and a matter of several studies. In our study, it was not found useful to identify patients at risk for cardiac dysfunction. All patients had normal baseline levels, and, during one year of trastuzumab treatment, in the majority of patients NT-proBNP level remained within normal level (less than 300 pg/mL). No patient showed a progressive increase. Nevertheless, we will probably have to wait for the results of clinical study CATS (Cardiotoxicity of Adjuvant Trastuzumab) to get more firm data on predictive value of this serum biomarker.^33^

Of the echocardiographic measurements, the diagnostic and predictive value of tissue Doppler imaging and its role in detecting cardiotoxicity in patients on trastuzumab therapy has so far not been studied extensively. In our study, we did not identify baseline peak systolic tissue Doppler velocity (Sm wave) as an important predictive factor of trastuzumab-related cardiotoxicity. Tissue Doppler velocities represent an easy measurable and reproducible alternative to LVEF to prediction early cardiac dysfunction in different cardiac diseases.^34^ Measurements are highly reproducible and reliable, and also less prone to intra- and inter-observer variability than any two-dimensional echocardiographic assessment of LVEF.^35^ Moreover, Sm wave velocity decline early in the course of trastuzumab therapy has been proposed as a marker of trastuzumab-related cardiac dysfunction that can be detected before a hemodynamic increase of cardiac volumes or a decrease in LVEF.^13,20^ In our study, we could also not demonstrate any baseline diastolic parameter (namely, LA area, Em wave and E/A ratio) as statistically significant predictive factors for trastuzumab-related cardiotoxicity. It is well known that diastolic dysfunction precedes systolic dysfunction, but may be present alone.^34^ Previous studies have yielded inconclusive and mixed results about a possible role of diastolic dysfunction as a predictor of anthracycline-related cardiotoxicity.^13,36^ Consequently, Doppler-derived diastolic indices are not deemed useful for early detection of anthracycline-related heart failure.^18^ With trastuzumab, Cochet et al.^16^ have reported that diastolic dysfunction (assessed by MUGA-derived time to peak rate of left ventricular filling) independently predicts trastuzumab-mediated cardiotoxicity. Conversely, in a small study with 42 patients on adjuvant trastuzumab treatment, early decline of tissue Doppler derived diastolic indices failed to predict trastuzumab-related cardiac dysfunction.^13^ Diastolic dysfunction indeed represents an early stage in the process of cardiac damage; it remains however present in late (systolic) stages as well. In our study, based on baseline diastolic function parameters, we could not identify patients with a LVEF decline of at least 10% from baseline during one year of adjuvant trastuzumab treatment. This is probably because all baseline values were in high-normal ranges. Due to enormous ability of cardiac reserves that have not been exhausted after a relative low cumulative dose of anthracyclines (patients in our study received from 300 to 400 mg/m^2^ epidoxorubicin or 240 mg/m^2^ doxorubicin), adaptive response has been able to fully compensate for toxicities and therefore over shaded predictive impact of otherwise very sensitive diastolic dysfunction indices. Although in our study we could not identify predictive value of any tissue Doppler derived measurements of either systolic or diastolic dysfunction, it is generally believed that serial measurements of tissue Doppler echocardiographic parameters (namely, Sm wave) could represent a feasible and reliable additional tool for early detection of threatening LVEF decline in patients receiving cardiotoxic oncologic therapy.^18^

In our study, only baseline systolic function emerged as the only independent statistically significant predictor of trastuzumab-related cardiac dysfunction. However, unexpected and contrary to the results of some previous studies with different cardiotoxicity definitions,^2,4,5^ we found that the risk was proportional and not inversely proportional to the patient's baseline LVEF; i.e. the chance of a LVEF decline ≥ 10% was significantly higher in patients with a higher baseline LVEF and the finding is therefore clinically not important. The average baseline LVEF of patients included in our study was high: 70.7% for those with a decline of 10% or more and 64.8% for those without it. In the randomized adjuvant clinical trials that used different cardiotoxicity definitions, a decline of 10% or 15% from baseline to below 50% or 55% was regarded as a cardiac event in asymptomatic patients. Looking at these trials, in all HERA^37,38^ and both American trials^2,5^ baseline LVEF was found as an important inversely proportional predictive factor for trastuzumab-related cardiotoxicity. In the American trial, patients with baseline LVEF ranging from 50% to 54% had 12-times higher risk compared to those with LVEF of 65% or more.^29^ Nevertheless, not all studies have shown baseline LVEF level to be important. In one of the first and biggest studies performed by Cardiac Review and Evaluation Committee including over 100 patients with proven trastuzumab-related cardiac dysfunction, baseline LVEF was not recognized as important predictor.^39^ Our patients, apart from HBP, had no cardiovascular disease, had normal heart function and were exposed to low doses of cardiotoxic anthracyclines, having, therefore, preserved high-normal LVEF. One possible explanation for an unexpected significantly higher chance of LVEF decline in patients with higher baseline LVEF could be in recruitment of compensatory mechanisms. It is well known that cardiomyocytes have a tremendous ability to preserve sufficient cardiac output. In patients with really high baseline LVEF, the LVEF decline of 10% of cardiac output is probably not compromised at all and adaptive response is activated with a delay. On the contrary, in patients with lower baseline LVEF, but still within normal range, adaptive response is activated earlier, thus further decline is opposed. The other possible explanation is that 10 percentage points represent a relatively higher value in patients with lower than higher baseline LVEF measurement. Nevertheless, according to the results of our study, a LVEF decline of ≥ 10% in patients with high-normal baseline level, although statistically significant, is not clinically relevant and predictive of cardiac dysfunction.

We have identified some limitations in our study. Our study employed standard transthoracic two-dimensional echocardiographic assessment. According to recommendations of ASE and EAE,^18^ echocardiography is suitable for serial evaluation of left ventricular structure and function in adult patients during and after cancer therapy; nevertheless, applying advanced echocardiographic methods, such as strain and strain-rate or the use of three-dimensional echocardiography, would strengthen the scientific accuracy of our results. However, standard echocardiography accompanied by tissue Doppler imaging used in our study is readily available in the clinical setting. Thus, our results provide wider applicability into the current clinical practice of cardio-oncology surveillance of breast cancer patients on adjuvant trastuzumab therapy. Additionally, our study was powered to detect a significant decline in LVEF, but not new-onset heart failure, which has almost a 5-fold lower incidence than asymptomatic cardiac dysfunction.^1^ We did not detect any case of new-onset heart failure; therefore it should be emphasized that this may be simply due to a small sample size.

## Conclusions

Identifying women at risk of developing trastuzumab-related cardiac dysfunction when starting adjuvant trastuzumab treatment continues to be an ongoing challenge. In this prospective cohort study, we have found that age, HBP, BMI, baseline LVEF, LVESV, Sm and Em wave, LA, E/A and NT-proBNP are not predictors of trastuzumab-related cardiac dysfunction in HER-2 positive breast cancer patients undergoing post-low dose anthracycline adjuvant trastuzumab therapy. In these patients, we found a high-normal baseline echocardiographically determined LVEF. A LVEF decline of ≥ 10% in patients with high-normal baseline level although statistically significant is not clinically relevant and predictive of cardiac dysfunction.
